# Topographical Visualization of the Reciprocal Projection between the Medial Septum and the Hippocampus in the 5XFAD Mouse Model of Alzheimer’s Disease

**DOI:** 10.3390/ijms20163992

**Published:** 2019-08-16

**Authors:** Sujin Kim, Yunkwon Nam, Yu-on Jeong, Hyun Ha Park, Seong-kyung Lee, Soo Jung Shin, Haram Jung, Byeong-Hyeon Kim, Sang Bum Hong, Yong Ho Park, Jihee Kim, Jaemin Yu, Doo-Han Yoo, Sun-Hyun Park, Seong Gak Jeon, Minho Moon

**Affiliations:** 1 Department of Biochemistry, College of Medicine, Konyang University, 158, Gwanjeodong-ro, Seo-gu, Daejeon 35365, Korea; 2 Department of Occupational Therapy, Konyang University, 158, Gwanjeodong-ro, Seo-gu, Daejeon 35365, Korea; 3 R&D Center for Advanced Pharmaceuticals & Evaluation, Korea Institute of toxicology, 141, Gajeong-ro, Yuseong-gu, Daejeon 34114, Korea

**Keywords:** Alzheimer’s disease, 5XFAD mice, medial septum, hippocampus, septo-hippocampo-septal loop, neural circuit, neural tracer, DiI, BDA

## Abstract

It is widely known that the degeneration of neural circuits is prominent in the brains of Alzheimer’s disease (AD) patients. The reciprocal connectivity of the medial septum (MS) and hippocampus, which constitutes the septo-hippocampo-septal (SHS) loop, is known to be associated with learning and memory. Despite the importance of the reciprocal projections between the MS and hippocampus in AD, the alteration of bidirectional connectivity between two structures has not yet been investigated at the mesoscale level. In this study, we adopted AD animal model, five familial AD mutations (5XFAD) mice, and anterograde and retrograde tracers, BDA and DiI, respectively, to visualize the pathology-related changes in topographical connectivity of the SHS loop in the 5XFAD brain. By comparing 4.5-month-old and 14-month-old 5XFAD mice, we successfully identified key circuit components of the SHS loop altered in 5XFAD brains. Remarkably, the SHS loop began to degenerate in 4.5-month-old 5XFAD mice before the onset of neuronal loss. The impairment of connectivity between the MS and hippocampus was accelerated in 14-month-old 5XFAD mice. These results demonstrate, for the first time, topographical evidence for the degradation of the interconnection between the MS and hippocampus at the mesoscale level in a mouse model of AD. Our results provide structural and functional insights into the interconnectivity of the MS and hippocampus, which will inform the use and development of various therapeutic approaches that target neural circuits for the treatment of AD.

## 1. Introduction

Alzheimer’s disease (AD), the most prevalent neurodegenerative disease, is characterized by memory loss, cognition impairments, and the progressive deposition of amyloid-β (Aβ) peptides and neurofibrillary tangles [1]. In particular, Aβ, which is believed to be a principal causative factor of AD pathogenesis, not only disrupts synaptic and circuit connections in the AD brain, but also abnormally accumulates 15 years before the onset of symptoms [2,3]. Indeed, Aβ burden is associated with the disruption of functional connectivity, even in clinically normal elderly populations [4,5,6]. Furthermore, Aβ-induced synaptic loss and neural network dysfunction have been well demonstrated in both patient and animal models of AD [7,8,9,10,11,12,13,14], and these alterations are ultimately associated with cognitive dysfunction in AD [15,16]. Therefore, the enhancement or protection of circuit integrity has been suggested as an effective strategy to treat cognitive decline in AD [17].

The medial septal complex, including the medial septum nucleus (MS) and diagonal band nucleus (DB), is known to play an important role in learning [18,19], short-term memory [20,21], and long-term memory [22]. The major axons of GABAergic, glutamatergic, and cholinergic neurons projected from the MS innervate the hippocampus through the fimbria/fornix [23,24,25]. In addition, the hippocampus not only receives inputs from the MS but also projects GABAergic and glutamatergic neurons back to the MS and lateral septum nucleus [25,26]. Furthermore, the interconnection between the MS and hippocampus constitute the septo-hippocampo-septal (SHS) loop, and the neurons in the two regions have a topographical and functional intercorrelation [26,27,28,29,30]. Due to this reciprocal connectivity, the hippocampal self-regulation of cholinergic input from the MS [23,31,32], the spatial representation of the hippocampus through activation of the MS [33], and memory formation in the hippocampus [25] are all manipulated by the SHS loop. Although the degeneration of the septo-hippocampal pathway has been reported in AD patients as well as AD animal models [10,34,35,36], there is no topographical study demonstrating the alterations in the SHS loop at the mesoscale level in the AD brain.

Investigation into the integration of brain connectivity is one of the main aims of neuroscience research. There are three levels of brain connectivity: macro-, meso-, and micro-scale. The macroscale connectome represents long-range pathways connecting patches of many nuclei [37]. The microscale connectome illustrates connectivity within an individual neuron [37]. Unfortunately, it is hard to determine the fiber termination positions in the gray matter at the macroscale level. In addition, at the microscale level, the small field of view limits its applicability to only a small fraction of the neural circuitry [38]. On the other hand, the mesoscale connectome provides a detailed understanding of the cell type construction of different brain regions and the patterns of afferent and efferent neurons that each of these cell types receives and forms. Moreover, the mesoscale connectivity bridges the information collected at the macroscale and microscale levels [39]. Furthermore, at the mesoscale connectome level, both long-range and local connections can be described using a sampling approach with diverse neuroanatomical tracers and can establish structural–functional integration [39,40]. Despite the importance of alterations in the bidirectional connection between the MS and hippocampus in AD, the topographical visualization of the SHS loop at mesoscale level has not yet been examined in an animal model of AD.

To evaluate the degeneration of the reciprocal connectivity between the MS and hippocampus in AD, we used five familial AD mutations (5XFAD) mice, which exhibit the main features of AD, such as accumulation of amyloid plaque, synaptic loss, neuroinflammation, and neuronal loss [41,42,43]. In patients with AD, the deposition of Aβ is regarded as the main pathologic feature, contributing significantly to the alteration of the synaptic network and neural circuit [9]. In addition, the brains of both AD patients and 5XFAD mice show Aβ deposition in the basal forebrain and hippocampus, along with Aβ-mediated synaptic degeneration and neural circuit loss [9,10,44]. Furthermore, AD patients have similar connectome properties to 5XFAD transgenic mice [45]. In the present study, we selected 4.5- and 14-month-old 5XFAD mice to identify connectivity between the MS and hippocampus before and after cognitive impairment [46,47].

We hypothesized that the SHS loop is altered in Aβ-overexpressing transgenic mice with synaptic and neuronal degeneration. To visualize first-order interconnectivity between the MS and hippocampus, we utilized non-transsynaptic retrograde/anterograde neural tracers and conducted a meticulous quantification.

## 2. Results

### 2.1. Aβ Accumulation in the MS and Hippocampal Formation of 5XFAD Mice

**Figure 1:**
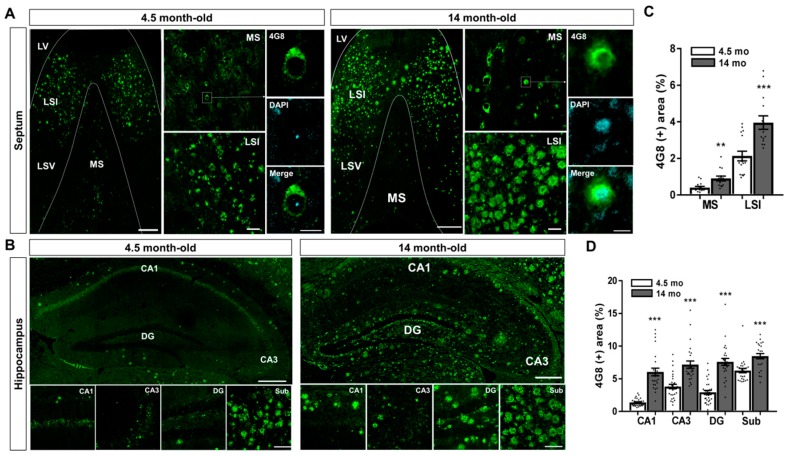
Amyloid-β (Aβ) deposition in the brain of the Aβ-overexpressing mice. (**A**,**B**) Visualization of Aβ accumulation in the MS and hippocampal formation by immunohistochemical staining with anti-4G8 antibody in brain sections of 4.5- and 14-month-old five familial AD mutations (5XFAD) mice. (**C**) Quantification of the 4G8 (+) areas in the MS and LSI of the 5XFAD mice. (**D**) Quantification of the 4G8 (+) areas in the CA1, CA3, DG, and Sub of the 5XFAD mice. ** *p* < 0.01 and *** *p* < 0.001 indicate significant differences between the 4.5- and 14-month old 5XFAD mice. Scale bar = 250 μm for hippocampus and septum; Scale bar = 40 μm for MS, LSI, CA1, CA3, DG, and Sub. Scale bar = 10 μm for MS. LV, lateral ventricle; LSI, lateral septal nucleus intermediate part; LSV, lateral septal nucleus ventral part oriens; MS, medial septal nucleus; DG, dentate gyrus; Sub, subiculum (n = 20–35 images/group).

The accumulation of Aβ plaques leads to the disruption of neuronal connectivity in the brain with AD [17]. To examine the Aβ-induced degeneration of the neural circuit in the brain, we chose 5XFAD mice which show similar Aβ accumulation to AD patients [45,48]. To characterize Aβ accumulation in the MS and hippocampal formation, we performed immunofluorescence staining in brain sections of both 4.5- and 14-month-old 5XFAD mice using the anti-4G8 antibody (Figure 1A,B). Histological data of Aβ staining showed that intracellular Aβ-accumulating cells were observed in the lateral septal nucleus intermediate part (LSI) from 4.5-month-old 5XFAD mice, while Aβ plaque deposition was present in the 14-month-old 5XFAD mice (Figure 1A). The 4G8 (+) area in the lateral septal nucleus intermediate part (LSI) significantly increased in the 14-month-old AD animal model compared with 4.5-month-old 5XFAD mice (Figure 1A,C). Interestingly, the MS showed a moderate accumulation of intracellular Aβ but no deposition of Aβ plaques in both 4.5- and 14-month-old 5XFAD mice (Figure 1A,C). Intracellular Aβ-accumulating cells were detected in the Sub (Figure 1B). On the other hand, 14-month-old 5XFAD mice significantly increased of the 4G8 (+) area in subregions of the hippocampal formation compared with 4.5-month-old 5XFAD mice (Figure 1B,D). These results demonstrate that the Aβ accumulation patterns in the brain of 5XFAD mice vary between the areas around the MS and hippocampal formation and at the different stages of AD progression.

### 2.2. Neuronal and Synaptic Degeneration in the MS and Hippocampal Formation of 5XFAD Mice

**Figure 2:**
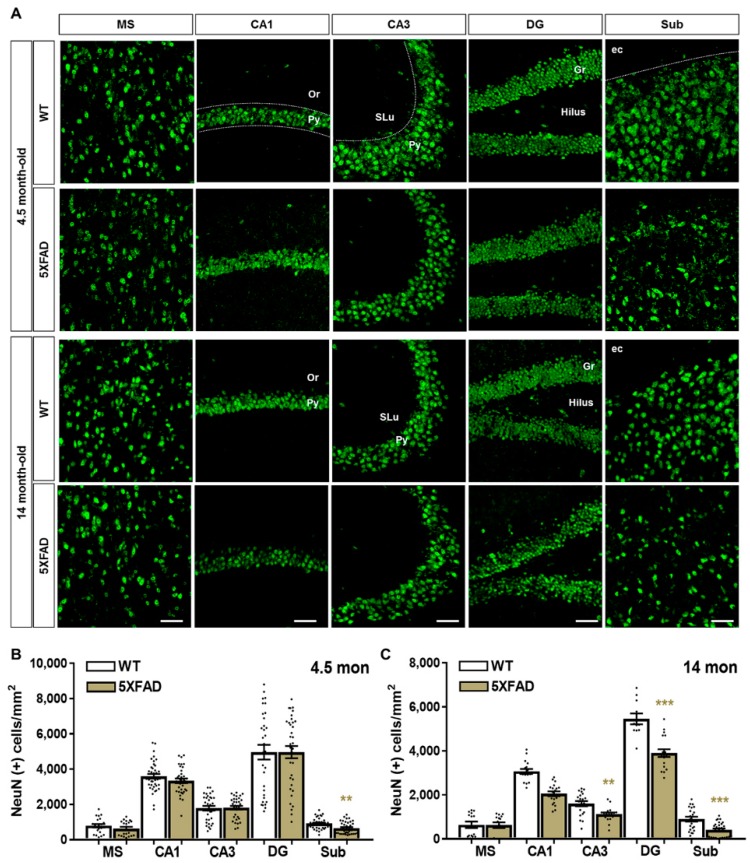
Neuronal loss in the MS and hippocampal formation of 5XFAD mice. (**A**) Neuronal nuclei (NeuN) were visualized using anti-NeuN antibody in the MS and hippocampal formation of wild-type (WT) and 5XFAD mice at 4.5 and 14 months of age. (**B**,**C**) The number of NeuN-positive cells per mm^2^ was calculated in the 4.5- and 14-month-old WT and 5XFAD mice. Scale bar = 100 μm. ** *p* < 0.01 and *** *p* < 0.001 indicate significant differences between the groups. Or, oriens layer; Py, pyramidal tract; SLu, stratum lucidum; Gr, granular layer; Mo, molecular layer; ec, external capsule (n = 20–35 images/group).

**Figure 3:**
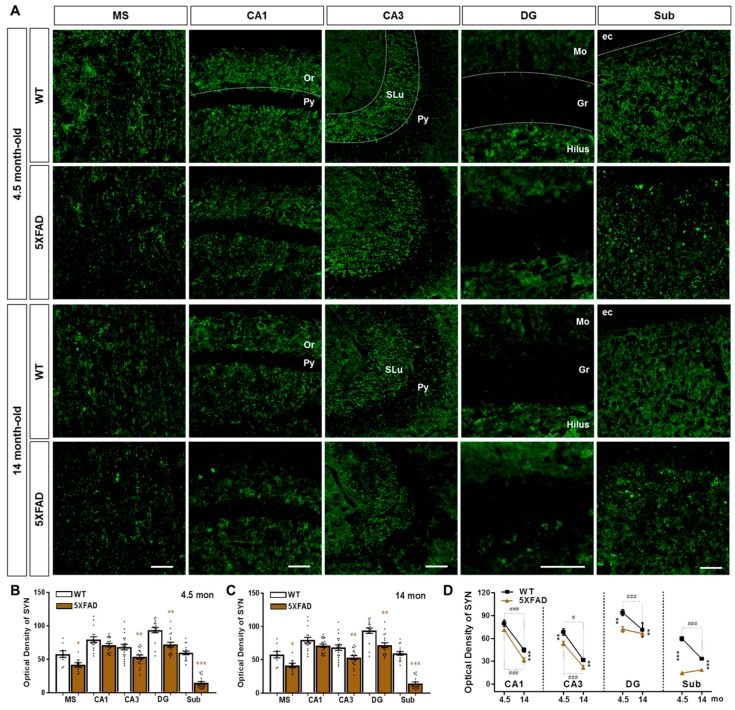
Synaptic degeneration in the hippocampal formation and MS of 5XFAD mice. (**A**) Pre-synaptic terminals were visualized using anti-synaptophysin (SYN) antibody in MS and the hippocampal formation of WT and 5XFAD mice at 4.5 and 14 months of age. (**B**–**D**) Fluorescence intensity of SYN immunoreactivity was quantified in the 4.5- and 14-month-old WT and 5XFAD mice. Scale bar = 100 μm. * *p* < 0.05, ** *p* < 0.01, and *** *p* < 0.001 indicate significant differences between the groups. ^#^ *p* < 0.05, and ^###^ *p* < 0.001 indicate significant differences between the different ages in the same group (n = 20–35 images/group).

It is well known that Aβ induces neuronal loss and synaptic degeneration [49], and that the loss of pre-synaptic protein synaptophysin occurs before the neuronal loss in the brains of AD patients [50]. To confirm that Aβ deposition is associated with neuronal and synaptic loss, we performed immunostaining to detect NeuN, a marker of neuronal nuclei (Figure 2A), and synaptophysin (SYN), a marker of pre-synaptic terminals (Figure 3A). The 4.5-month-old 5XFAD mice showed significant neuronal loss compared to wild-type (WT) mice in the Sub (Figure 2B), which displayed robust accumulation of intracellular Aβ in 5XFAD mice (Figure 1B,D). In 14-month-old 5XFAD mice, however, the Sub as well as the CA3 and DG regions exhibited significantly decreased numbers of NeuN-positive cells compared to WT mice; but there was no significant decrease in the MS and CA1 (Figure 2C). On the other hand, the optical density of SYN was significantly decreased not only in the Sub but also in MS, CA3, and DG, which did not exhibit significant neuronal loss in 4.5-month-old 5XFAD mice (Figure 2B and Figure 3B–D). All regions observed showed significant neuronal loss in 14-month-old 5XFAD mice compared to WT mice (Figure 3B–D). Notably, we observed age-related synaptic loss in all regions (Figure 3D). These results successfully confirm the previous findings that synaptic degeneration precedes neuronal cell death in the brain with Aβ deposition and that the significant synaptic loss occurs with aging and AD progression.

### 2.3. Neuroanatomical Tracing of the Hippocampo-Septal Pathway Using a Retrograde Tracer

**Figure 4:**
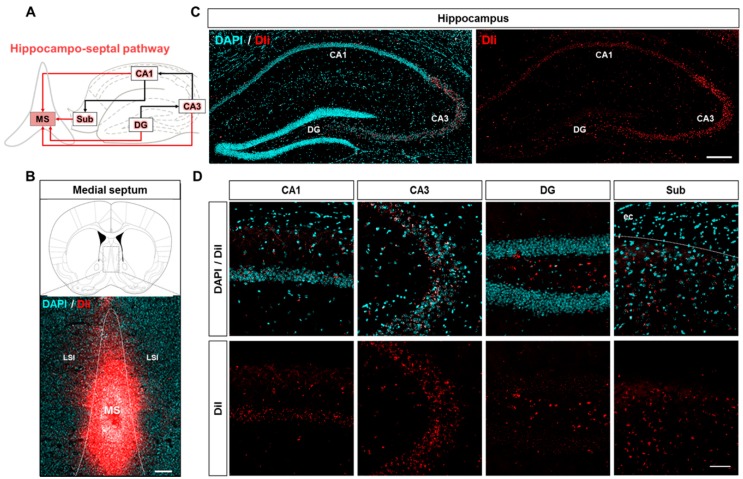
Profiling of the hippocampo-septal pathway with the retrograde neuronal tracer DiI. (**A**) Schematic of the hippocampo-septal pathway. Red arrows indicate the afferent projections from the hippocampal formation to the MS. Black arrows indicate the indirect afferent projections from the hippocampal formation to the MS. (**B**) Verification of the MS coordinates by stereotaxic injection of the retrograde tracer DiI. Scale bar = 200 μm. (**C**) Visualization of DiI fluorescence retrogradely transported from the MS to the hippocampus. Scale bar = 200 μm. (**D**) Magnified images represent DiI-positive somata in the subregions of the hippocampal formation. DAPI was used to counterstain the nuclei. Scale bar = 100 μm.

Before analyzing the hippocampo-septal pathway in the Aβ-overexpressing transgenic mice, we first visualized the well-described projections from the hippocampal formation to the MS in WT mice. The previous studies have well shown the hippocampo-septal pathway in the brain (Figure 4A) [25,26,29,30,51]. To visualize the hippocampo-septal pathway, we performed stereotaxic injection of the retrograde tracer DiI into the MS of WT mice (Figure 4B). Four days after the injection, the DiI-positive afferent neurons projecting to the MS were observed in the hippocampal formation, including the CA1, CA3, DG, and Sub (Figure 4C,D). These results validate that the retrograde tracer DiI can be used to visualize the hippocampo-septal pathway by showing the well-characterized projections from the hippocampus to the MS.

### 2.4. Disruption of the Hippocampo-Septal Pathway in 5XFAD Mice

**Figure 5:**
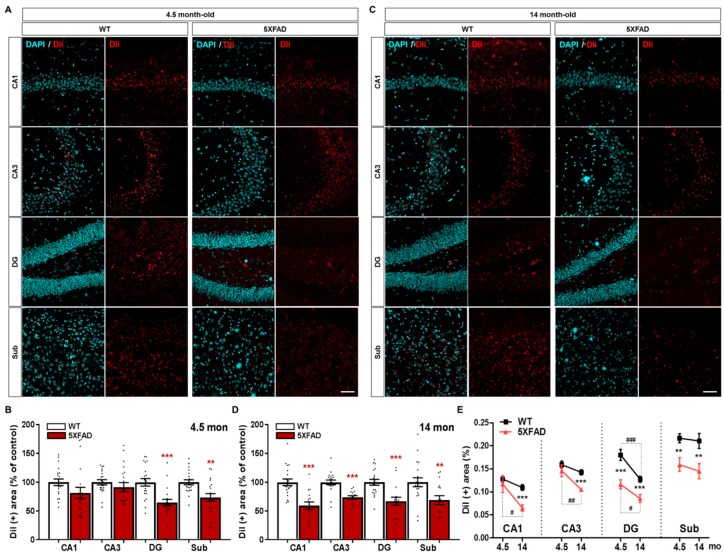
Degeneration of the hippocampo-septal pathway in WT and 5XFAD mice. (**A**) Representative images of DiI-containing somata in the CA1, CA3, DG, and Sub of WT and 5XFAD mice at the age of 4.5 months. (**B**) Quantification of the DiI-positive area of the hippocampal formation of 4.5-month-old WT and 5XFAD mice. (**C**) Representative images of DiI-containing somata in the CA1, CA3, DG, and Sub of WT and 5XFAD mice at the age of 14 months. (**D**) Quantification of the DiI-positive area of the hippocampal formation of 14-month-old WT and 5XFAD mice. (**E**) Comparison of DiI-positive area in the hippocampo-septal degeneration by aging and AD progression. Scale bar = 100 μm. ** *p* < 0.01, *** *p* < 0.001 indicate significant differences between the WT and 5XFAD mice of the same age. ^#^ *p* < 0.05, ^##^ *p* < 0.01, and ^###^ *p* < 0.001 indicate significant differences between the different ages in the same group (n = 20–35 images/group).

Recent studies have reported that the connectome property of 5XFAD mice parallels that of patients with AD [45,48]. To examine whether the hippocampo-septal pathway is altered in AD brains, we performed the stereotaxic injection of DiI into the MS of 5XFAD mice and WT mice. In 4.5-month-old 5XFAD mice, which exhibit no cognitive deficits [52], there was a statistically significant decrease in DiI-labeled area in the DG and the Sub compared to WT mice, but not in the CA1 or CA3 (Figure 5A,B,E); however, 14-month-old 5XFAD mice showed a marked reduction in DiI-positive areas in all subregions of the hippocampal formation, including CA1 which showed no significant neuronal loss compared to WT mice (Figure 5C–E). Furthermore, a comparison of the DiI-positive areas between 4.5-month-old and 14-month-old mice showed a significant age-related reduction only in the DG in WT mice, whereas 5XFAD mice showed a significant decrease in the CA1, CA3, and DG (Figure 5E). These results suggest that septal afferents from the DG and Sub are markedly disrupted in the AD brain even before the onset of cognitive decline (Figure 5B,D,E), implying the impairment of the hippocampo-septal pathway during the progression of AD as well as normal aging.

### 2.5. Neuroanatomical Tracing of the Septo-Hippocampal Pathway Using an Anterograde Tracer

**Figure 6:**
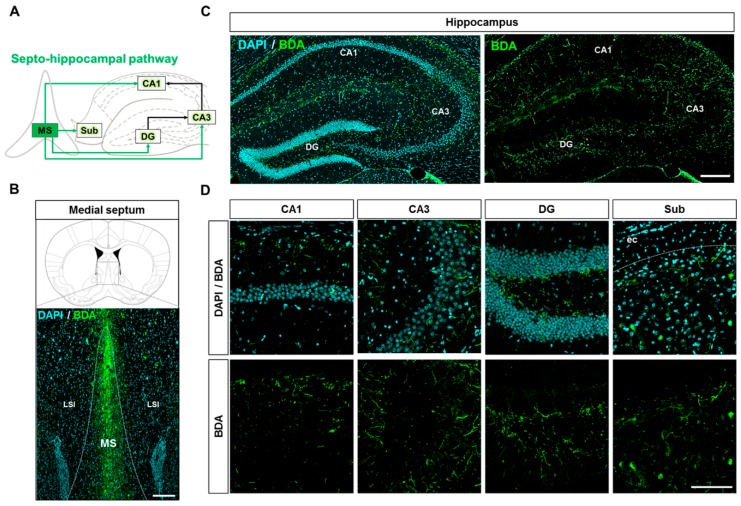
Profiling of the septo-hippocampal pathway with the anterograde neuronal tracer BDA. (**A**) Schematic of septo-hippocampal pathway. Green arrows indicate the efferent projections from the MS to the hippocampal formation. Black arrows indicate the indirect efferent projections from the MS to the hippocampal formation. (**B**) Verification of the MS coordinates by stereotaxic injection of the anterograde tracer BDA. Scale bar = 200 μm. (**C**) Visualization of BDA anterogradely transported from the MS to the hippocampus. Scale bar = 200 μm. (**D**) Magnified images represent BDA-labeled axon terminals in the subregions of the hippocampal formation. DAPI was used to counterstain the nuclei. Scale bar = 50 μm.

Prior to examining the septo-hippocampal pathway in AD brains, we first confirmed the efferent projections from the MS to the hippocampal formation in WT brains based on the previous reports (Figure 6A) [10,19,25,26,29,34,35,51,53]. To visualize the septo-hippocampal pathway, we performed stereotaxic injection of the anterograde tracer BDA into the MS of WT mice (Figure 6B). Six days after the injection, BDA-labeled axon terminals were detected in the CA1, CA3, DG, and Sub (Figure 6C,D). These results indicate that the anterograde tracer BDA can visualize the septo-hippocampal pathway by showing well-known projections from the MS to the hippocampal formation.

### 2.6. Disruption of the Septo-Hippocampal Pathway in 5XFAD Mice

**Figure 7:**
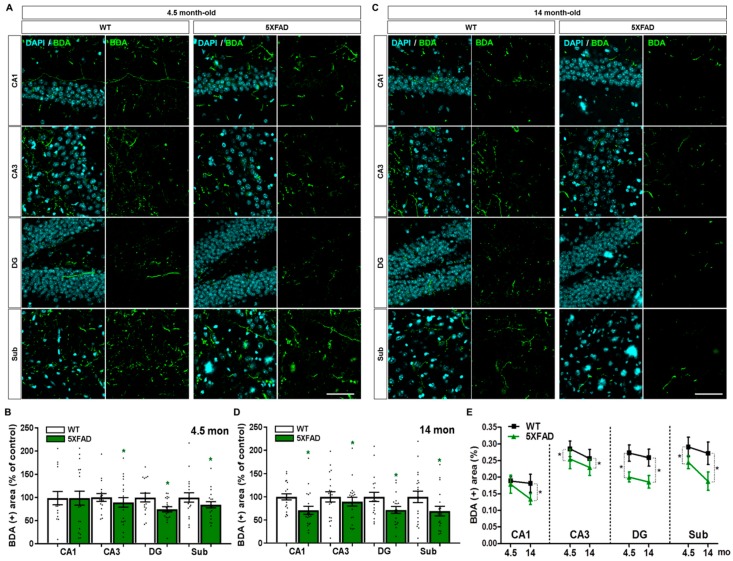
Degeneration of the septo-hippocampal pathway in WT and 5XFAD mice. (**A**) Representative images of BDA-labeled axon terminals in the CA1, CA3, DG, and Sub of WT and 5XFAD mice at the age of 4.5 months. (**B**) Quantification of the BDA-positive area in the hippocampal formation of 4.5-month-old WT and 5XFAD mice. (**C**) Representative images of BDA-labeled axon terminals in the CA1, CA3, DG, and Sub of WT and 5XFAD mice at the age of 14 months. (**D**) Quantification of the BDA-positive area in the hippocampal formation of 14-month-old WT and 5XFAD mice. (**E**) Comparison of BDA-positive area in the septo-hippocampal degeneration by aging and AD progression. Scale bar = 100 μm. * *p* < 0.05 indicates significant differences between the WT and 5XFAD mice of the same age (n = 20–35 images/group).

Although the septo-hippocampal pathway is known to be impaired in brains with AD, the direct topographical changes are not fully understood during the pathogenesis of AD [10,19,34,35]. To investigate whether the septo-hippocampal pathway is altered in AD animal models, we performed stereotaxic injection of BDA into the MS of 5XFAD mice and WT mice at 4.5 and 14 months of age. Consistent with the results of synaptic degeneration (Figure 3B), the 4.5-month-old 5XFAD mice showed significantly reduced BDA-labeled areas in the CA3, DG, and Sub compared to WT mice (Figure 7A,B,E). The 14-month-old 5XFAD mice also exhibited significant reduction in BDA-positive areas in all subregions of the hippocampal formation compared to WT mice (Figure 7C–E). Furthermore, the comparison of BDA-positive areas between 4.5- and 14-month-old mice displayed a trend towards degeneration with age throughout the hippocampal formation in both 5XFAD and WT mice (Figure 7E). These results indicate that efferents from the MS to the CA3, DG, and Sub are significantly damaged in the AD brain even in the early stage of disease (Figure 7B,D,E), and that septo-hippocampal connectivity further deteriorates with the progression of AD.

## 3. Discussion

The aim of this study was to identify topographical changes in the reciprocal projections between the MS and hippocampus during AD pathogenesis. Consistent with previous studies [50], our findings revealed that the Aβ-induced neural circuit disruption is accompanied by synaptic impairment and precedes the neuronal loss in the brain. In addition, the current findings are the first to (1) visualize the bidirectional interconnection between the MS and hippocampus, and (2) show alterations in the SHS loop at the mesoscale level in the 5XFAD brain. The MS and hippocampus are interconnected to form the SHS loop which is known to regulate the numerous cognitive functions, such as recognition, learning, and memory [25,26,29,54]. Based on cholinergic hypothesis in AD, although connectivity between the MS and hippocampus has been established in AD patients at the macroscale level, the mesoscale connectome of the SHS loop has not yet been elucidated during disease progress in the AD brain [55]. To investigate the SHS loop at the mesoscale level in AD brain with aging, we conducted stereotaxic injection of the anterograde tracer BDA or retrograde tracer DiI into the MS of 4.5- and 14-month-old 5XFAD and WT mice. Subsequently, we visualized and quantified the AD-related pathology and DiI- and BDA-positive axon terminals and cells in the brains of 5XFAD and WT mice.

**Figure 8:**
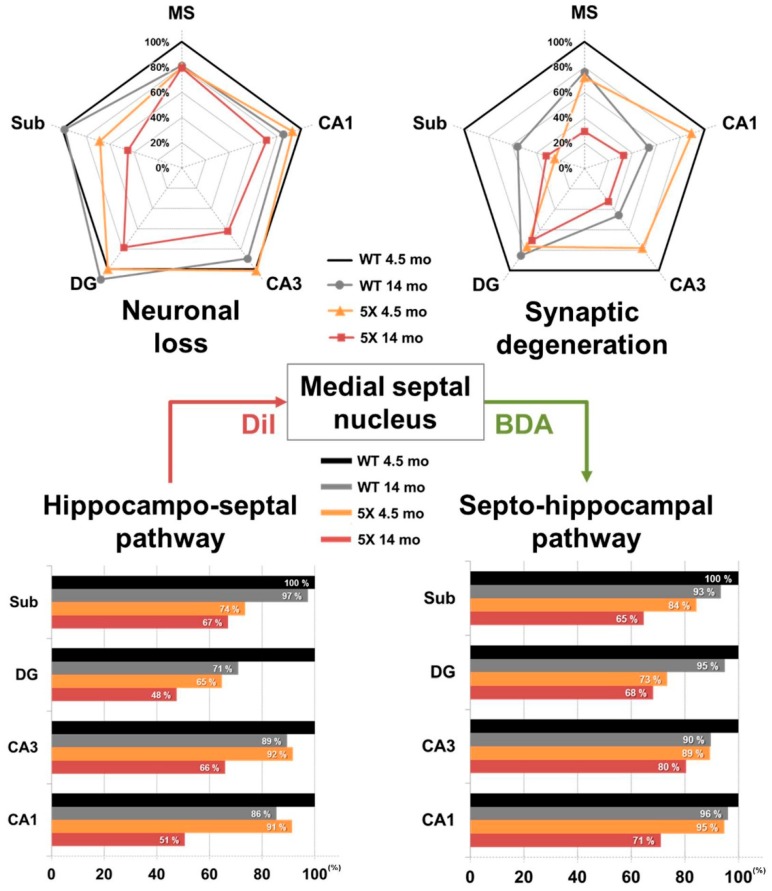
Schematic of the septo-hippocampo-septal (SHS) loop alterations in 5XFAD mice. The histological results in the MS and hippocampal formation were normalized to those of 4.5-month-old WT mice.

By visualizing brain sections with immunofluorescence against SYN, we found that 4.5-month-old 5XFAD mice exhibited a significant reduction in pre-synaptic terminals in the MS, CA3, DG, and Sub, while the 14-month-old 5XFAD mice showed decreased pre-synaptic terminals throughout the entire hippocampal formation (Figure 3). This demonstrates that the 5XFAD mice display age-dependent robust synaptic degeneration in the hippocampal formation before neuronal loss as compared to WT mice (Figure 2 and Figure 3). Surprisingly, 4.5-month-old 5XFAD mice, which show no cognitive dysfunction, exhibited decreased DiI-positive area in the DG and the Sub (Figure 5A,B) and decreased BDA-positive areas in the CA3, DG, and Sub (Figure 7A,B). Furthermore, 14-month-old 5XFAD mice showed a reduction in DiI- and BDA-positive areas throughout the hippocampal formation. In brief, there were three significantly impaired connections: the circuit between the MS and DG, the reciprocal projections between the MS and Sub, and the pathway from the MS to CA3 (Supplementary Figure S2). Moreover, the circuit degeneration in 14-month-old 5XFAD mice was aggravated compared to 4.5-month-old 5XFAD mice. Particularly, there were significant decreases in the DiI-positive areas in the CA1, CA3 and DG of 14-month-old 5XFAD mice compared 4.5-month-old 5XFAD mice (Figure 5E). Therefore, the septo-hippocampal and hippocampo-septal pathways constituting the SHS loop had begun to deteriorate in 4.5-month-old 5XFAD mice before the onset of cognitive impairment (Figure 5E and Figure 7E). Furthermore, degradation of the SHS loop connectivity was concurrent with synaptic loss (Figure 3B–D) and was observed prior to significant neuronal loss (Figure 2B,C). These results suggest that the degeneration of the SHS loop is aggravated in an age-dependent manner in the CA1, CA3, and DG of 5XFAD mice. In addition, the projections from the DG to the MS in WT mice were significantly decreased with aging (Figure 5E). To summarize the results of the present study, an integrated diagram is presented in the Figure 8 for the reciprocal projection, synaptic degeneration, and neuronal loss between the MS and hippocampus in 5XFAD mice.

The AD brain displays altered functioning of cortical circuits, including changed patterns of synchronous activity, and, in particular, a serious deficit in cholinergic septo-hippocampal innervation [56]. In addition, Aβ is known to preferentially accumulate in excitatory neurons (glutamatergic and cholinergic neurons) in the AD brain [57,58]. Furthermore, significant cholinergic neuronal loss of the MS was demonstrated in the 6-month-old 5XFAD mice with cognitive dysfunction [46]. Moreover, local injections of Aβ into the MS could cause the impairment of both cholinergic and glutamatergic septal projections; however, GABAergic neurons were intact after septal injection of Aβ [59]. In postmortem AD brains, Aβ was found in glutamatergic boutons that displayed fewer dendritic spines and less severe neuronal damage [60]. Furthermore, MS rhythmicity, which is known to underlie memory consolidation, was decreased by Aβ [61]. Moreover, soluble oligomers of Aβ and protofibrils have been shown to cause cholinergic dysfunction, presumably resulting in age-related memory decline [62]. Based on cell-type specific vulnerability against Aβ, it can be speculated that there might be Aβ-vulnerable circuits/connectivity between the MS and hippocampus, such as interconnections between the MS and DG/Sub.

This study sets the stage for future investigations to identify the topographical structure of cell type-specific circuits, such as cholinergic, glutamatergic, and GABAergic circuits, in the MS and the hippocampus, utilizing cell type-specific tracers to observe cell type-specific alterations of the SHS loop. In addition, targeting the AD-vulnerable circuits demonstrated in the present study would enable the investigation of the roles of specific circuits or therapeutic strategies. Activation or inhibition of the specific circuits can be achieved using optogenetic approaches [63], deep brain stimulation [64], transcranial direct current stimulation [65], transcranial magnetic stimulation [66,67], and theta-burst microstimulation [68]. In summary, the results from this study demonstrate that (1) interconnections between the MS and DG/Sub as well as projections from MS to CA3 are significantly impaired in the early stages of AD pathogenesis in the 5XFAD mice; (2) degeneration of the SHS loop occurs prior to neuronal loss and cognitive dysfunction in the 5XFAD brain; (3) disruption of the SHS loop is accelerated during the progression of AD; (4) the SHS loop in the healthy brain shows a trend toward age-related degeneration; and (5) the rate of impairment of the hippocampo-septal circuit in the 5XFAD brain is more severe than that of the WT brain. Taken together, our data highlight the reciprocal connections between the MS and hippocampus impaired during the progression of AD. Our results also provide structural and functional insights into various therapeutic approaches for the treatment of AD, including those targeting neural circuits.

## 4. Materials and Methods

### 4.1. Animals

The 4.5- and 14-month-old transgenic mice with five familial AD mutations (5XFAD) express three mutations in the human *APP* gene (Florida^V717I^, Swedish^K670N/M671L^, and London^I716V^) and two mutations in the human *PSEN1* gene (L286V and M146L). The animals were obtained from The Jackson Laboratory (#006554; Bar Harbor, ME, USA). 5XFAD transgenic mice were identified by genotyping, and littermates were used as WT controls. Five mice were assigned to each experimental group. Male mice were used for the experiments at 4.5 months of age, and a mix of female (n = 2) and male (n = 3) mice were used for the experiments at 14 months of age. It has been shown that young female 5XFAD mice exhibited slightly higher Aβ_42_ levels than age-matched male 5XFAD mice, however, this increase diminished at older ages [42]. For stereotaxic surgery, the animals were deeply anesthetized by intraperitoneal injection of Avertin (Tribromoethanol; Sigma-Aldrich, St. Louis, MO, USA) at a dose of 250 μg/kg. Maintenance and treatment of the animals were performed in accordance with the principles of the Care and Use of Laboratory Animals (NIH Publication No. 85-23, revised 1985) and the Animal Care and Use Guidelines of Konyang University (Project code: P-18-06-A-01; August 1st, 2018).

### 4.2. Stereotaxic Injection of DiI for Retrograde Tracing

The 1,1′-dioctadecyl-3,3,3′,3′-tetramethyl-indocarbocyanine perchlorate (DiI; Sigma-Aldrich) was dissolved in dimethyl sulfoxide (DMSO) to a concentration of 20 mM. The stock solution was diluted to 82 μM with phosphate-buffered saline (PBS). DiI was injected into the MS at 0.5 µL/min for 3 min. Stereotaxic coordinates were as follows: AP, +0.62 mm; ML, 0.00 mm; DV, −4.00 and −4.50 mm from the bregma. After DiI injection, the needle was gently withdrawn, and the skin was sutured. Four days after surgery, the animals were anesthetized and perfused with 4% paraformaldehyde in 0.1 M phosphate buffer (PB) (Supplementary Figure S1).

### 4.3. Stereotaxic Injection of BDA for Anterograde Tracing

Biotinylated dextran amine (BDA; 10,000 MW; Molecular Probes Inc., Eugene, OR, USA) was dissolved in 0.01 M PB to a concentration of 10% (*w*/*v*). The 10% BDA was injected into the MS at 1.5 µL/min for 3 min. Stereotaxic coordinates were as follows: AP, +0.62 mm; ML, 0.00 mm; DV, −4.00 and −4.50 mm from the bregma. After BDA injection, the needle was gently withdrawn, and the skin was sutured. Six days after surgery, the animals were anesthetized and perfused with 4% paraformaldehyde in 0.1 M PB (Supplementary Figure S1).

### 4.4. Brain Tissue Preparation

Four days after the DiI injections and six days after the BDA injections, the animals were anesthetized, transcardially perfused with 0.05 M PBS, and then fixed with cold 4% paraformaldehyde in 0.1 M PB. Next, brains were removed and post-fixed in 0.1 M PB containing 4% paraformaldehyde for 20 h at 4 °C and subsequently immersed in 30% sucrose in 0.05 M PBS solution for 3 days at 4 °C for cryoprotection. The brains were embedded with Surgipath^®^ frozen section compound (Leica Biosystems, Wetzlar, Germany) and cut into serial 30-μm-thick coronal sections with a CM1850 cryostat (Leica Biosystems). The tissue sections were stored in a cryoprotectant (25% ethylene glycol, 25% glycerol, and 0.05 M PB) at 4 °C until further analysis.

### 4.5. Immunofluorescence Labeling

Four or seven coronal sections were selected from each mouse from the septal and hippocampal region with reference to Paxinos and Franklin’s *The Mouse Brain in Stereotaxic Coordinates* [69] (Supplementary Figure S3). To examine the immunoreactivity of Aβ, neuronal nuclei (NeuN), and synaptophysin (SYN), free-floating sections were incubated overnight at 4 °C with the mouse anti-4G8 antibody (1:2000; Cat.# 800701, BioLegend, San Diego, CA, USA), mouse anti-NeuN antibody (1:100; Cat.# MAB377, Merk KGaA, Darmstadt, Germany), or mouse anti-SYN antibody (1:500; Cat.# S5768, Sigma-Aldrich), respectively, in blocking solution (0.05% bovine serum albumin, 1.5% normal goat serum and 0.3% Triton X-100 in PBS) [70]. After washing three times for five minutes in PBS, the sections were incubated with goat Alexa 488-conjugated anti-mouse IgG (1:200; Cat.# A11001, Thermo Fisher Scientific Inc., Waltham, MA, USA) or goat Alexa 594-conjugated anti-mouse IgG (1:200; Cat.# A11005, Thermo Fisher Scientific Inc.) for 1 h at room temperature. The tissue sections were mounted on ProbeOn™ Plus Microscope Slides (Thermo Fisher Scientific Inc.) and coverslipped with Fluoroshield™ with DAPI (Sigma-Aldrich) to counterstain the nuclei.

### 4.6. Image Acquisition and Analysis

To trace the DiI-labeled cells and BDA-labeled axon terminals, the entire tissue sections were imaged with a Zeiss LSM 700 Meta confocal microscope (Carl Zeiss AG, Oberkochen, Germany). Then, the DiI-labeled cells and BDA-labeled axons were quantified from 20–35 representative images containing the hippocampus using ImageJ software (NIH, Bethesda, MD, USA) as follows: (1) images were converted to 8 bit for image quantification; (2) following conversion, images were thresholded for the area of DiI-labeled cells or BDA-labeled axons, and background signals were removed; (3) topographic anatomical area of the hippocampal formation containing CA1, CA3, dentate gyrus (DG), and subiculum (Sub) were designated based on DAPI counterstaining; (4) thresholded images for the designated brain area were quantified by the “Analyze particles” tool for the “% Area” value of the DiI-labeled cells or BDA-labeled axons; (5) values from two groups (WT and 5XFAD) were normalized to the controls using the following equations: % of control = (%Area_WT or 5XFAD_/%Area_average of WT_) × 100. The quantification of 4G8 (+) area% is the same as the above process, and the quantification of NeuN (+) cells per area is derived by dividing the positive cell number derived in (4) into the designed area (Supplementary Figure S4A–D).

### 4.7. Statistical Analysis

Quantified histological data were processed for statistical analysis with GraphPad Prism 7 (Systat Software, La Jolla, CA, USA). Values are represented as the mean ± standard error of the mean. Data were analyzed with independent *t*-tests or Mann–Whitney test for differences between the two groups (Supplementary Figure S5). Differences with a *p*-value less than 0.05 were considered statistically significant. Image acquisition, histological quantification, and statistical analysis were performed in a blind manner for each group.

## Supplementary Materials

Supplementary materials can be found at https://www.mdpi.com/1422-0067/20/16/3992/s1.

## Abbreviations

5XFAD	five familial AD mutations	
AchE	acetylcholine	
AD	Alzheimer’s disease	
Aβ	amyloid-β	
BDA	biotinylated dextran amine	
DB	diagonal band nucleus	
DG	dentate gyrus
DiI	1,1′-dioctadecyl-3,3,3′,3′-tetramethyl-indocarbocyanine perchlorate
DMSO	dimethyl sulfoxide
LSI	lateral septal nucleus intermediate part
LSV	lateral septal nucleus ventral part oriens
MS	medial septum
nBM	nucleus basalis magnocellularis
NeuN	neuronal nuclei
PB	phosphate buffer
PBS	phosphate-buffered saline
SHS	septo-hippocampo-septal;
Sub	subiculum
SYN	synaptophysin
WT	wild-type
