# Supplementary material for: Topographical Visualization of the Reciprocal Projection between the Medial Septum and the Hippocampus in the 5XFAD Mouse Model of Alzheimer’s Disease
Source: Int J Mol Sci. 2019 Aug 16;20(16):3992. doi: 10.3390/ijms20163992 (PMC6721212; doi:10.3390/ijms20163992)
Supplement: Supplementary file 1 [file ijms-20-03992-s001.pdf]

Supplementary Figures

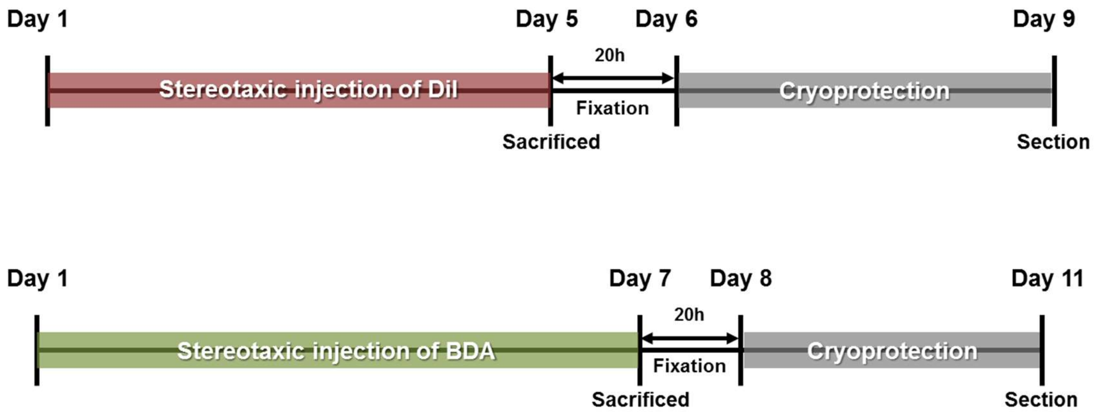

Supplementary Figure 1. The experimental design for examining the altered SHS loop in 5XFAD mice brain. DiI and BDA were injected into the MS of 5XFAD mice. The tracer was taken up by axon terminals/cell bodies within the MS and then transported to the hippocampus through the axon of the neurons. Histochemical analyses were conducted either four or six days after injection to visualize the SHS loop.

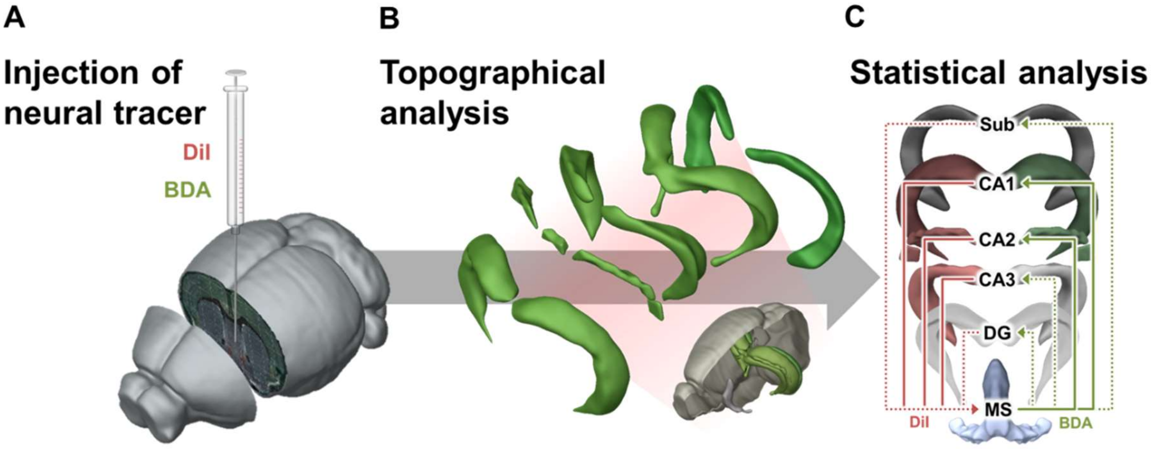

Supplementary Figure 2. The experimental design for investigating the septo-hippocampo-septal loop in an animal model of AD. **A)** DiI and BDA were injected into the MS of WT and 5XFAD mice. **B)** Histochemical analyses were performed to examine and quantify the number of DiI- and BDA-labeled axon terminals and somata. **C)** Schematic of the degenerated reciprocal connectivity between the MS and the hippocampus in the brain of 4.5-month-old 5XFAD mice. The disrupted pathways are shown by dotted arrows, while solid arrows indicate intact projections.

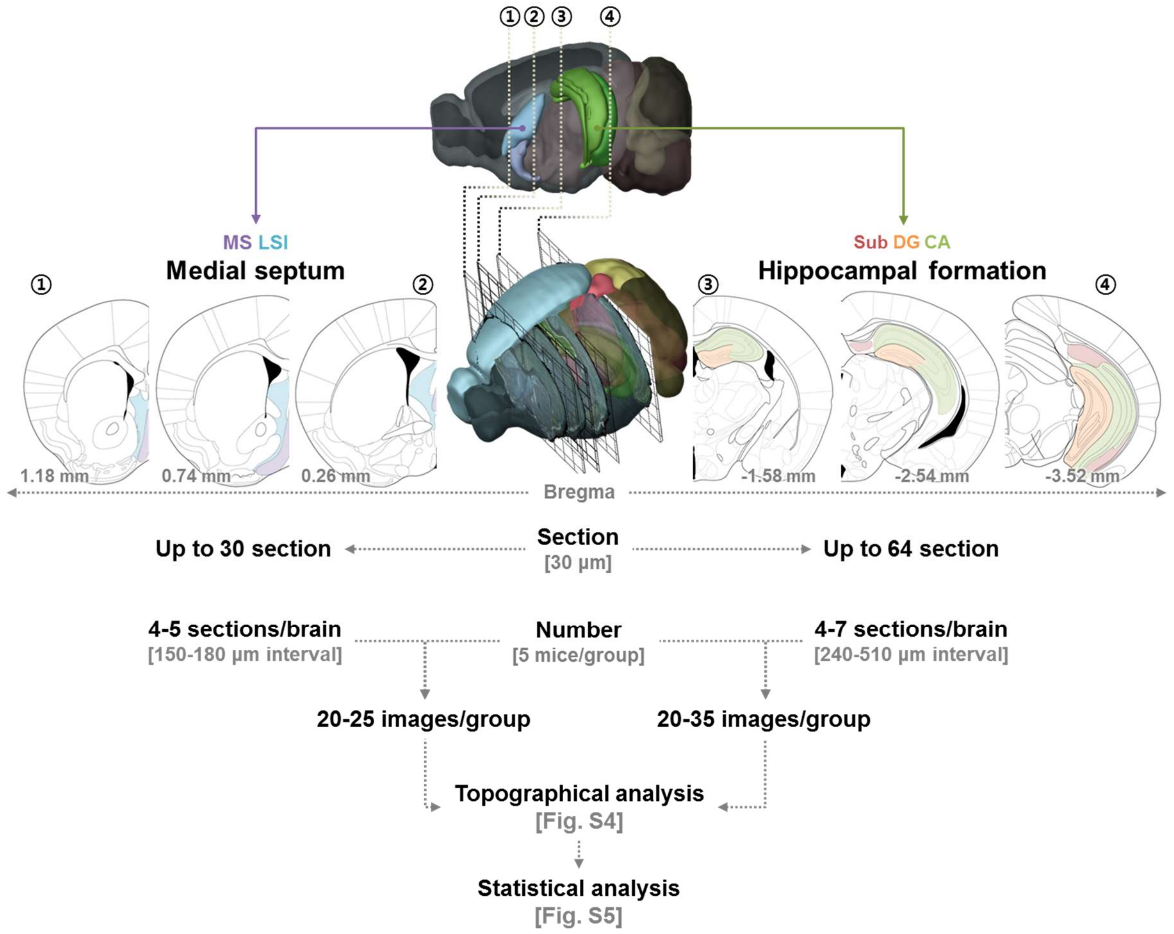

Supplementary Figure 3. Preparation of mice brain section for immunohistochemistry. From ① to ②, septal area was designated from 1.18 mm to 0.26 mm from the bregma. From ③ to ④, hippocampal area was designated from -1.58 mm to -3.52 mm from the bregma. Fixed and cryoprotected mouse brains were coronally sectioned at a thickness of 30 μm in cryostat. Thirty (septal region) or sixty-four (hippocampal region) sections per mouse were obtained using the cryosection. In the septal area, four to five sections per brain were taken from five mice at 150-180 μm intervals to obtain 20-25 images for MS and LSI. In the hippocampal area, four to seven sections per brain were taken from five mice at 240-510 μm intervals to obtain 20-35 images for CA1, CA3, DG, and Sub. The acquired images were subjected to topographical quantification and statistical analysis in a blind manner.

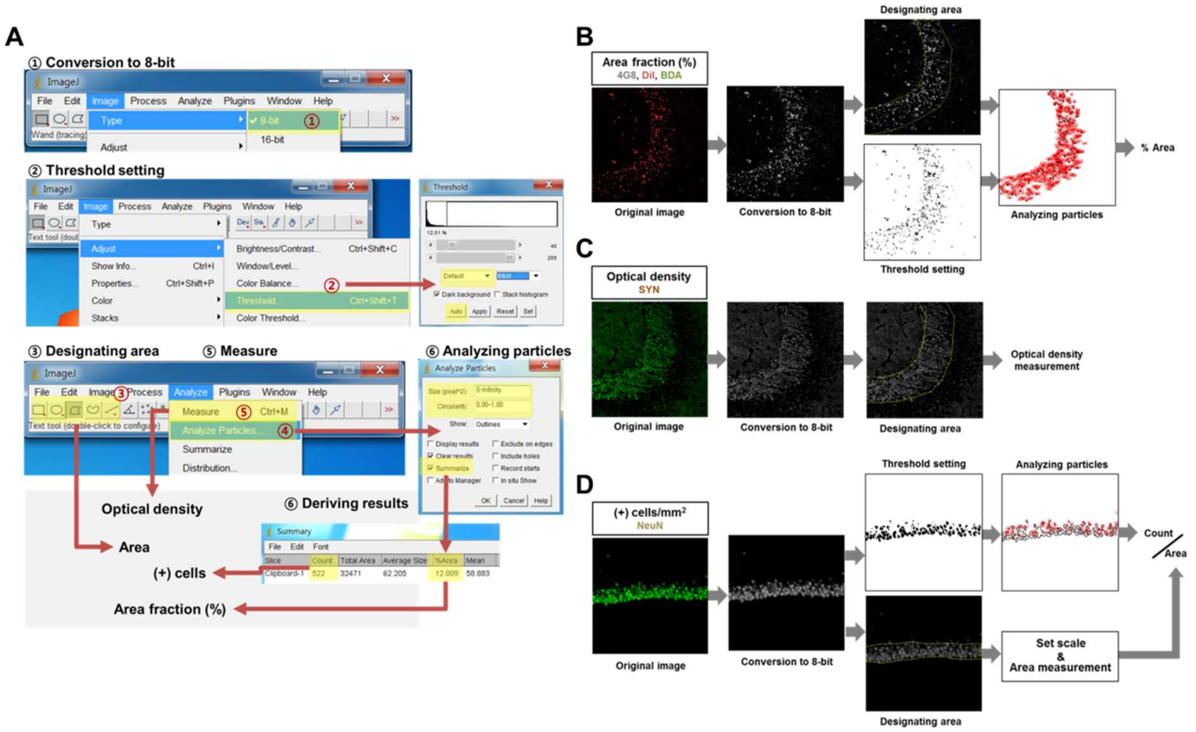

Supplementary Figure 4. The topographical analysis procedure for quantification of fluorescent signals in the mice brains. **A)** The procedures for the analysis steps for "area faction", "optical density", and "cells per area" in the ImageJ program. **B)** Quantitative procedure of "area fraction" applied for histological analysis of fluorescent signals of 4G8, DiI, and BDA. **C)** Quantitative procedure of "optical density" applied for histological analysis of SYN-immunoreactivity. **D)** Quantitative procedure of "positive cells/area" applied for histological analysis of NeuN-immunoreactivity.

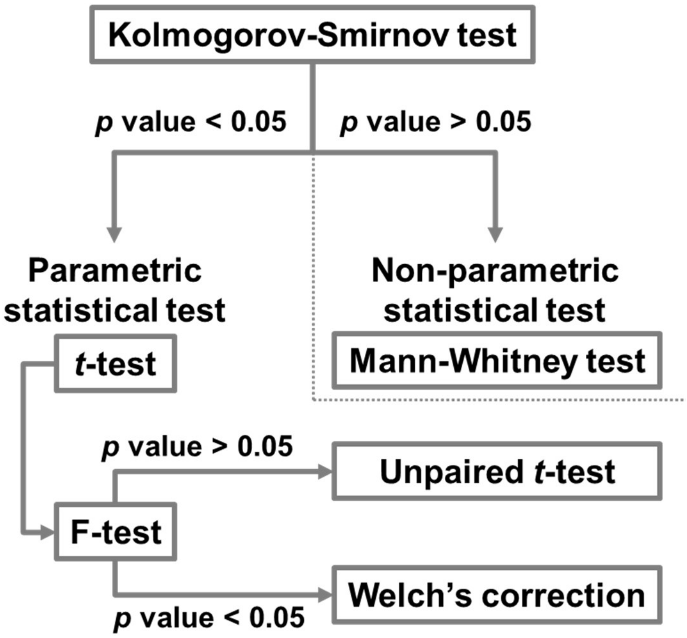

Supplementary Figure 5. Statistical analysis of the data from topographical quantification of positive signals. To verify the statistical significance between WT and 5XFAD mice, Kolmogorov-Smirnov tests were applied to classify whether to apply parameter statistical test (Student's *t*-test) or nonparametric statistical test (Mann-Whitney test). In case of parameter statistics, the variance homoscedasticity was analyzed via F-test. The unpaired t-test was applied when the variances of the two groups were homogeneity of variance, and Welch's correction was applied for the heteroscedasticity of variance.
